# Effects of Acute Aerobic Exercise and Task Repetition on the Neural Correlates of Executive Function in Young and Older Adults: A Crossover fMRI Study

**DOI:** 10.1002/jmri.70329

**Published:** 2026-06-10

**Authors:** Ryota Asahara, Marina Fukuie, Daisuke Hoshi, Junyeon Won, Rong Zhang, Jun Sugawara, Takashi Tarumi

**Affiliations:** ^1^ Integrated Research Center for Self‐Care Technology National Institute of Advanced Industrial Science and Technology (AIST) Ibaraki Japan; ^2^ Health and Medical Research Institute National Institute of Advanced Industrial Science and Technology (AIST) Ibaraki Japan; ^3^ Human Informatics and Interaction Research Institute National Institute of Advanced Industrial Science and Technology (AIST) Ibaraki Japan; ^4^ Department of Sports Sciences Japan Institute of Sports Sciences Tokyo Japan; ^5^ Institute for Exercise and Environmental Medicine Texas Health Presbyterian Hospital Dallas Dallas Texas USA; ^6^ Department of Neurology University of Texas Southwestern Medical Center Dallas Texas USA; ^7^ Department of Internal Medicine University of Texas Southwestern Medical Center Dallas Texas USA; ^8^ Department of Biomedical Engineering University of Texas Southwestern Medical Center Dallas Texas USA; ^9^ Institute of Health and Sport Sciences University of Tsukuba Ibaraki Japan

**Keywords:** acute aerobic exercise, aging, brain activity, executive function, randomized crossover study, task repetition

## Abstract

**Background:**

Acute aerobic exercise may alter neural recruitment supporting executive function; however, age‐related differences and task repetition effects remain unclear.

**Purpose:**

To investigate the effects of acute aerobic exercise and task repetition on brain activity during task switching in young and older adults.

**Study Type:**

Prospective randomized crossover study.

**Population:**

Seventeen young and nineteen older healthy adults (18 females).

**Field Strength/Sequence:**

3.0 T, BOLD fMRI.

**Assessment:**

fMRI was conducted during a task‐switching paradigm before and at 15 and 45 min following 30 min of moderate‐intensity walking or sitting on separate days. Neural and behavioral switch costs were computed as differences in BOLD activation, reaction time, and error rates between switch and nonswitch trials.

**Statistical Tests:**

Linear mixed‐effects model examined BOLD signals and behavioral performance. Repeated measures correlation examined within‐subject associations. *p* < 0.05 was considered statistically significant.

**Results:**

Older adults exhibited significantly greater task‐related BOLD activation in the bilateral superior frontal and middle temporal gyri, right angular gyrus, and left precuneus than young adults, despite slightly lower performance (*p* ≤ 0.002). Task repetition significantly reduced activation in the left middle occipital and middle frontal gyri and right cerebellar cortex in both groups, whereas reaction time significantly improved only in older adults. Following walking, activation in the right dorsolateral prefrontal cortex remained stable in older adults but significantly decreased in young adults, without corresponding behavioral changes (*p* ≥ 0.365). Regardless of age, activation in the right postcentral gyrus and bilateral superior parietal lobules remained stable after walking but significantly decreased after sitting. Reduced activation with task repetition was significantly associated with faster reaction time, particularly in older adults.

**Data Conclusion:**

Acute aerobic exercise may preserve regional brain activation without measurable behavioral benefits, whereas task repetition reduces brain activation across age groups, suggesting modest neural effects of acute aerobic exercise on executive function.

**Level of Evidence:**

2.

**Technical Efficacy Stage:**

2.

## Plain Language Summary

This study examined how a single bout of 30‐min moderate‐intensity walking affects brain activity during a cognitive task in young and older adults. Participants completed brain MRI scans while performing a task that required switching between rules, before and after walking or sitting. The results showed that walking helped maintain brain activity in regions involved in task switching, although task performance did not improve. Repeating the task reduced brain activity and improved performance, especially in older adults. These findings suggest that acute aerobic exercise may support brain function, highlighting the importance of considering both age and repetition effects in studies.

## 1 Introduction

Acute aerobic exercise may transiently enhance cognitive performance in healthy adults and clinical populations [1, 2, 3, 4], although the underlying neural mechanisms are incompletely understood. Despite mixed findings across individual studies [5, 6, 7, 8, 9, 10, 11, 12], meta‐analyses have demonstrated small but significant improvements in cognitive performance, with larger effects observed in older adults and individuals with lower baseline cognitive performance [1, 2, 3]. Meta‐analytic evidence also suggests that moderate‐intensity aerobic exercise elicits greater improvements in cognitive performance than exercise at light or vigorous intensity [13].

Executive function, defined as higher‐level cognitive control processes that enable goal‐directed behavior [14, 15], has frequently been examined as a target of acute exercise interventions [3]. Specifically, cognitive flexibility, a component of executive function reflecting the ability to switch between tasks in response to changing environmental demands [16], is supported by a distributed fronto‐parietal network, including the lateral prefrontal cortex, inferior parietal lobule, and posterior inferior temporal lobes [17].

Functional MRI (fMRI) is a valuable imaging tool for measuring task‐related blood oxygenation level‐dependent (BOLD) signals, reflecting the neurovascular coupling between neural activity and local hemodynamic response [18, 19]. Compared with other neuroimaging techniques (e.g., functional near‐infrared spectroscopy and electroencephalography), fMRI provides higher spatial resolution that enables the assessment of neural activation across widespread cortical and subcortical regions, despite higher acquisition costs and susceptibility to motion artifacts [20]. Prior fMRI studies suggest that acute moderate‐intenisty aerobic exercise may modulate task‐related brain activation in both young and older adults [20, 21, 22, 23, 24, 25, 26], with increased activation in frontal and parietal regions (e.g., inferior, superior, and middle frontal gyri and inferior parietal gyrus), and may decrease activation in the anterior cingulate cortex during executive function tasks [21, 22, 23, 24, 26]. However, whether and how age, task repetition, and sex together modulate the acute exercise effects on task‐related BOLD activation remains unclear.

Age has been shown to modify task‐related BOLD activation patterns, with older adults often exhibiting greater or more widespread neural recruitment during cognitive tasks [27, 28]. A previous study has also indicated age‐related differences in the hemodynamic response function (HRF) [29], suggesting changes in neurovascular coupling with age. In addition, a recent systematic review of neuroimaging studies has reported sex differences in executive control, with males and females potentially engaging distinct neural strategies depending on task demands [30]. These age‐ and sex‐related differences may modulate the neural effects of acute aerobic exercise, highlighting the importance of study designs that enable direct comparisons across these groups within the same experimental framework.

Most previous fMRI studies investigating acute exercise effects have relied solely on post‐intervention measurements, omitting pre‐intervention assessments [20]. Although such designs can reduce protocol complexity and scan duration, the absence of baseline measurements limits the ability to account for day‐to‐day variability in cognitive performance and brain activation [3, 20, 31]. Moreover, repeated exposure to the same cognitive task may induce learning effects, which typically improve cognitive performance and may reduce task‐related BOLD activation even without any intervention [32, 33, 34, 35]. Therefore, to mitigate these limitations, a within‐subject crossover design with pre‐ and post‐intervention assessments may control for inter‐individual variability and learning effects [3]. Furthermore, statistical adjustment for baseline data enables exercise‐induced changes in cognitive performance and BOLD activation that can be assessed relative to condition‐specific pre‐intervention reference measures, thereby accounting for potential baseline shifts and day‐to‐day variability [31].

The primary aim of this study was to examine the acute effects of aerobic exercise on task‐related BOLD activation during a cognitive flexibility task in healthy young and older adults using a within‐subject crossover design with pre‐ and post‐intervention assessments. We hypothesized that a single bout of 30‐min moderate‐intensity walking would increase regional BOLD activation during task switching in older adults, accompanied by improved behavioral performance. A secondary aim was to examine the effects of task repetition on brain activation and cognitive performance. We hypothesized that task repetition would reduce BOLD activation while improving performance, and that these effects would differ by age. An additional aim was to examine whether reductions in BOLD activation are associated with improved processing efficiency using within‐subject correlation analyses. Finally, we explored sex differences in the effects of acute exercise and task repetition on brain activation, given evidence for sex‐related variation in executive control [30].

## 2 Methods

### 2.1 Ethics Statement

All experimental procedures were approved by the local Institutional Review Board of the National Institute of Advanced Industrial Science and Technology (HF2023‐873) and conducted in accordance with the Declaration of Helsinki and the Belmont Report. Written informed consent was obtained from all participants.

### 2.2 Participants

**FIGURE 1:**
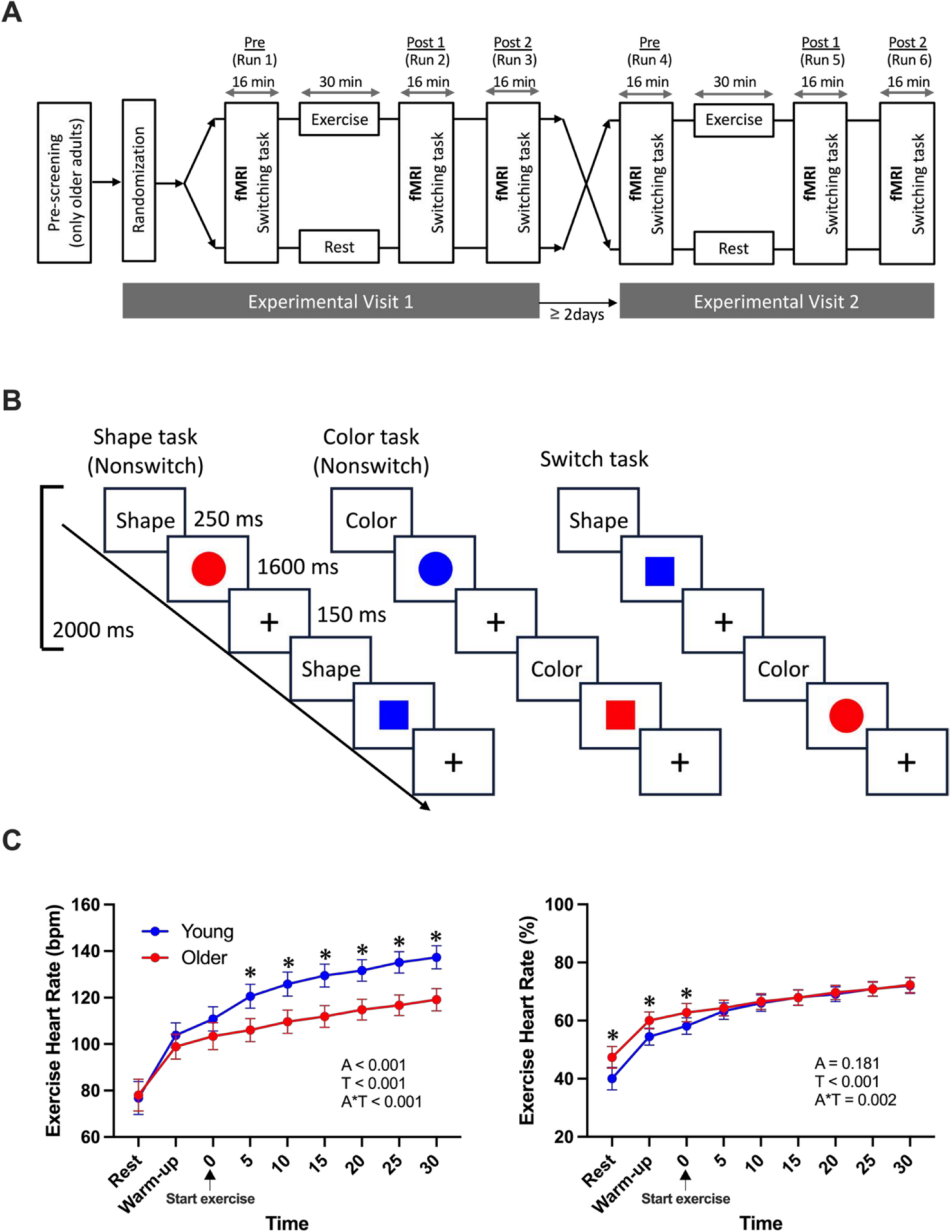
(A) Experimental protocol illustrating the randomized crossover, pre‐ and post‐intervention design. (B) Task‐switching paradigm. Nonswitch and switch tasks were alternated pseudo‐randomly. (C) Changes in absolute (left) and relative (right) heart rate responses to moderate‐intensity walking in young (blue) and older (red) adults. Relative heart rate was calculated as a percentage of age‐predicted maximal heart rate. Values represent estimated marginal means with 95% confidence intervals and *p* values from linear mixed models. * indicates *p* < 0.05 versus young at the same time point. A, age; T, time.

Seventeen healthy young adults (8 women; 25 ± 2 years) and 19 older adults (10 women; 62 ± 5 years) participated in the study. Participants were recruited via community flyers, newspaper advertisements, and local websites. For the older group, an initial telephone interview screened candidates for subjective cognitive complaints, history of major clinical conditions, habitual exercise participation, and availability for three study visits (one for in‐person pre‐screening and two for experimental sessions; see Figure 1A). During pre‐screening (Visit 1), candidates were excluded if they met any of the following criteria: (1) diagnosis of psychiatric or neurological disorders (including dementia) or use of medications with cognitive side effects, (2) Mini‐Mental State Examination (MMSE) score < 26, (3) smoking within the past 5 years, (4) MRI contraindications (e.g., metal implants), or (5) diagnosis of hypertension or diabetes including the use of antihypertensive and blood glucose lowering medications. Blood pressure (BP) was measured at least twice at pre‐screening using an automated sphygmomanometer (BP‐900; Tanita, Tokyo, Japan) and participants with systolic BP > 140 mmHg and/or diastolic BP > 90 mmHg were excluded. Exercise‐related health risks were evaluated based on medical history, habitual exercise participation, and blood pressure assessments to ensure that participants could safely perform the exercise intervention.

### 2.3 Experimental Protocol

A within‐subject, counter‐balanced crossover design was used to minimize between‐subject variability, intervention order effects, and task repetition bias (Figure 1A). To control for time‐of‐day influences and mitigate potential practice effects, both experimental visits were conducted at the same time of day for each participant and separated by at least 48 h (mean interval = 12.4 ± 10.8 days). Eight young (47%) and nine older (47%) participants were randomized to the exercise condition on the first experimental visit, while the remainder began with the rest condition. Participants were instructed to fast for at least 2 h and refrain from caffeine, alcohol, and vigorous physical activity for at least 24 h prior to each visit. At each visit, participants had three fMRI scans during a cognitive task: before (pre), ~15 min after (post 1; mean = 14.8 ± 4.2 min), and ~45 min after (post 2; mean = 47.3 ± 4.3 min) the intervention (exercise or rest). Participants remained inside the scanner between the post 1 and post 2 scans. In total, six fMRI scans were acquired per participant across the two experimental visits. Task repetition effects were assessed by analyzing all six consecutive runs regardless of exercise and rest conditions.

On the first experimental visit, participants provided informed consent and were randomized to either exercise or rest condition (Figure S1). Height and body mass were measured using a stadiometer and scale, and seated blood pressure and heart rate were measured using an automated cuff (HEM‐7130; Omron Corporation, Kyoto, Japan). Prior to the pre‐intervention measurements on each experimental day, participants completed at least 10 min of practice to familiarize themselves with the fMRI task in a room adjacent to the MRI suite. In the exercise condition, participants walked < 3 min to an exercise physiology laboratory in the same building as the MRI room and performed 35 min of treadmill walking (5‐min warm‐up +30‐min moderate‐intensity walking). Exercise intensity was set at 50%–70% of age‐predicted maximal heart rate calculated as 208–0.7 × age [36]. Heart rate was continuously monitored during walking using the three‐lead electrocardiogram telemetry system (Life Scope BSM‐3400, Nihon Kohden, Tokyo, Japan), while treadmill speed and grade were adjusted to maintain the target heart rate. After exercise, participants rated their perceived exertion using the Borg scale [37] and then returned to the MRI room for post‐intervention fMRI scans. In the rest condition, participants remained seated in a chair for 30 min in a room adjacent to the MRI room. No specific cognitive task or reading materials were provided to maintain a resting baseline. Participants were instructed to stay awake and to refrain from excessive conversation during the rest condition, while study staff monitored arousal levels and prevented dozing.

### 2.4 Task Switching

Cognitive flexibility, a core component of executive function [14], was assessed with a nonverbal perceptual task‐switching paradigm administered during fMRI scanning [17]. Participants performed three task types: two nonswitch tasks (shape and color) and one switch task (Figure 1B). In the shape task, the cue “shape” appeared and participants judged whether the stimulus was a circle or a square, ignoring its color. In the color task, the cue “color” appeared and participants judged whether the stimulus was red or blue, ignoring its shape. In the switch task, the cue indicated which attribute (shape or color) to judge, requiring participants to alternate between rules. Responses were made with the right index or middle finger using an MRI‐compatible fiber optic button box (Current Designs, Philadelphia, PA, USA). Participants were instructed to respond as quickly and accurately as possible.

Stimuli were presented centrally on a screen using PsychoPy (Open Science Tools Ltd., Nottingham, UK). The instruction cue (“shape” or “color”) was presented for 250 ms and was followed by a 1600 ms target stimulus and a 150 ms fixation cross. Reaction time and error rate were recorded for nonswitch and switch tasks. Reaction time and error rate‐based switch cost were defined as the difference between switch and nonswitch tasks and served as the primary behavioral measures of cognitive flexibility [16]. It was calculated by subtracting the mean reaction time or error rate of nonswitch tasks from that of switch tasks.

Each experimental session lasted 16 min and included 24 task blocks (8 shape, 8 color, 8 switch) and 24 fixation blocks. Each block lasted 20 s. Task blocks followed a pseudo‐random sequence, with shape, color, and switch blocks occurring at equal frequency (e.g., shape‐color‐switch‐switch‐color‐shape‐shape‐switch‐color). Nonswitch blocks contained 10 repeated trials of the same task (e.g., shape‐shape‐shape‐shape), while switch blocks contained 10 trials with trial types alternating pseudo‐randomly every 1–2 trials (e.g., color‐shape‐shape‐color). Each switch block comprised 6 switch and 4 nonswitch trials. Task instructions and a 10‐min practice session with partial task exposure were provided at both experimental visits, with additional guidance as needed.

### 2.5 MRI Data Acquisition

MRI data were collected using a 3‐Tesla scanner (Ingenia, Philips Medical Systems, Best, the Netherlands) with a 32‐channel head coil (dStream Head). Headphones were used to minimize scanner noise and facilitate communication. Visual stimuli were projected onto a screen and viewed via a mirror mounted on the head coil. MRI‐compatible goggles were provided if needed. Following localizer scans, BOLD images were acquired using a gradient echo‐planar imaging (EPI) sequence with the following parameters: field of view (FOV) = 230 × 230 mm^2^, 50 axial slices (no gap), voxel size = 3 × 3 × 3 mm^3^, repetition time (TR) = 2000 ms, echo time (TE) = 35 ms, flip angle = 90°, and scan duratio*n* = 16 min. Higher‐resolution structural images were also acquired using a T1‐weighted magnetization‐prepared‐rapid‐gradient‐echo (MPRAGE) sequence with: FOV = 240 × 240 mm^2^, 170 sagittal slices (no gap), voxel size = 1 × 1 × 1 mm^3^, TR = 8 ms, TE = 3.7 ms, flip angle = 8°, and scan duration = 5 min and 38 s. Structural scans were acquired on the rest condition day.

### 2.6 MRI Data Processing

Preprocessing was conducted using Statistical Parametric Mapping (SPM12; Wellcome Department of Imaging Neuroscience, London, UK). The first five volumes of each EPI scan were discarded to account for T1 equilibration effects. EPI images were slice‐timing corrected and realigned to the first functional volume. MPRAGE images were co‐registered to the functional images, segmented, and spatially normalized to the Montreal Neurological Institute (MNI) template. Functional data were smoothed using an 8‐mm full width at half maximum Gaussian kernel and high‐pass filtered (cutoff = 0.0078 Hz). Subject‐level analysis used a general linear model (GLM) with regressors for each task block: 8 shape (nonswitch), 8 color (nonswitch), and 8 switch. Each task was modeled using the canonical hemodynamic response function (duration = 20 s) with its time derivative. The temporal derivative was used to account for potential variability in the latency of the hemodynamic response among young and older participants, thereby reducing potential bias in the estimation of age effects. An implicit masking threshold of 0.2 was applied to avoid exclusion of low‐intensity voxels near cortical boundaries. Motion parameters (six rigid‐body transformations) were included as nuisance covariates. Three contrasts were generated per participant: switch > nonswitch, switch > fixation, and nonswitch > fixation. Group‐level analysis focused on the switch > nonswitch contrast, representing the neural switch cost. Whole‐brain voxelwise analysis was performed within the SPM framework to assess the acute exercise, age, and task repetition effects.

Predefined region‐of‐interest (ROI) analysis was conducted to examine the effects of acute exercise on task‐related BOLD activity. According to the literature [17, 22, 24, 26], the bilateral dorsolateral prefrontal cortex (DLPFC), ventrolateral prefrontal cortex (VLPFC), and supramarginal gyrus (SMG) were selected as a priori ROIs. These ROIs were defined as 5‐mm radius spheres centered at MNI coordinates: left DLPFC [−46, 26, 28], right DLPFC [44, 24, 26], left VLPFC [−48, 22, 6], right VLPFC [45, 20, 0], left SMG [−40, −60, 42], and right SMG [42, −56, 38] using Marsbar toolbox. Percent BOLD signal changes were extracted from each ROI at pre, post 1, and post 2 for both exercise and rest conditions and averaged across all voxels within each sphere. The extracted ROI's BOLD signal data were subjected to linear mixed‐effects model (LMM) analysis to examine the acute exercise, age, and task repetition effects. Additional ROI analysis was performed on significant clusters identified in the whole‐brain analysis. This ROI analysis was conducted to aid interpretation of the direction and temporal BOLD signal changes. Regions showing significant effects of task repetition were used to define spherical ROIs centered at the peak MNI coordinates. Percent BOLD signal changes were extracted from the ROIs and analyzed using LMMs to further characterize the age and repetition effects. Total brain volume was calculated from MPRAGE images using FreeSurfer 7.2.0 (https://surfer.nmr.mgh.harvard.edu/) and normalized by intracranial volume (%ICV) to account for head size.

### 2.7 Sample Size Estimates

Sample size was estimated using the “simr” package in R v4.4.2 (R Foundation for Statistical Computing, Vienna, Austria) by conducting a simulation‐based power analysis for linear mixed‐effects models. Because no previous fMRI studies have employed a comparable pre–post crossover design to examine the acute effects of exercise, effect size assumptions were informed by prior studies comparing post‐exercise and post‐rest brain activation in older adults [26]. This study reported greater BOLD activation following acute moderate‐intensity aerobic exercise across several cortical regions, with a median effect size of approximately Cohen's *d* = 0.5. Assuming a moderate effect size (*d* = 0.5) for the exercise‐induced change in BOLD activation, power analysis was performed using the “powerSim” function with 100 simulations. The sample size was iteratively increased using the “powerCurve” function until statistical power reached 80%. This procedure indicated that 30 participants were required to detect the condition × time interaction effect under the assumed parameters (two‐sided α = 0.05, target power = 0.80).

### 2.8 Statistical Analysis

Age and task repetition effects on BOLD activation were first examined using whole‐brain voxelwise analysis. A flexible factorial design was implemented to test main and interaction effects of age and repetition, with family‐wise error (FWE) correction applied for multiple comparisons. Subsequently, BOLD signals were extracted from significant clusters, displayed over six consecutive runs regardless of randomization, and analyzed by LMM, including age (young vs. older) and repetition (six consecutive runs) as fixed factors. Behavioral performance was also analyzed by LMM with the same fixed factors. Models included a random intercept, compound symmetry covariance structure, and Bonferroni‐corrected post hoc comparisons. Effect sizes for interaction effects in the LMM analysis were quantified using partial eta squared (η_*p*_^2^), and Cohen's *d* was calculated for post hoc pairwise comparisons.

Exercise effects were then tested with adjustment for pre‐intervention data collected on each experimental day. For the primary analysis, LMM included condition (exercise vs. rest), time (pre, post 1, post 2), and age as fixed factors, with BOLD data extracted from the predefined ROIs and behavioral performance as dependent variables. A complementary whole‐brain voxelwise analysis was performed using a flexible factorial design with the main and interaction effects of condition, time, and age, with FWE correction applied for multiple comparisons. BOLD data extracted from significant clusters were further analyzed by LMM adjusted for pre‐intervention values.

To explore whether acute exercise and repetition effects on BOLD activation differed by sex, whole‐brain voxelwise analyses with two separate flexible factorial designs were tested, including the main and interaction effects of sex, condition, and time, and those of sex and repetition.

Within‐subject associations between BOLD activation and behavioral performance were assessed using repeated‐measures correlation [38, 39]. Demographic and exercise data were compared with independent‐samples *t*‐tests or Chi‐square tests. Statistical significance was set at *p* < 0.05. Whole‐brain results were thresholded at *p* < 0.001 (uncorrected) and considered significant at cluster‐level FWE‐corrected *p* < 0.05. Analyses were performed using SPM12, SPSS v25 (IBM, Armonk, NY), and R v4.4.2.

## 3 Results

Among the 1 49 older adults screened, 30 were excluded because of elevated blood pressure (*n* = 16), medical conditions (*n* = 5), MRI contraindications (*n* = 5), or unspecified reasons (*n* = 4) (Figure S1). Of the 19 older adults who enrolled, two withdrew after the first experimental visit. Thus, statistical analyses were performed on 16 young and 19 older participants.

### 3.1 Participant Characteristics

Table 1 shows participant characteristics. Body height (*p* = 0.091), mass (*p* = 0.060), and index (*p* = 0.187) did not differ significantly between young and older groups. Total brain volume was significantly smaller in older adults. Systolic (*p* = 0.086) and diastolic BP (*p* = 0.490) and heart rate (*p* = 0.356) did not differ significantly between groups. During walking, the treadmill speed required to reach target heart rate was significantly faster in young adults than in older adults, while grade did not differ significantly (*p* = 0.666). Figure 1C shows heart rate responses during exercise in each age group. Although the absolute increase in heart rate was significantly greater in young participants, both groups walked at 60%–70% of the age‐predicted maximal heart rate (*p* = 0.479). Rate of perceived exertion during exercise did not differ significantly between groups (*p* = 0.568).

**TABLE 1:** Characteristics of young and older groups.

	Young	Older	*p*
	(*n* = 16)	(*n* = 19)	
Women (n, %)	8 (50%)	10 (53%)	0.877
Age (years)	25 ± 2	62 ± 5	< 0.001
Height (cm)	167 ± 9	162 ± 7	0.091
Body mass (kg)	62 ± 9	56 ± 10	0.060
Body mass index (kg/m^2^)	22 ± 3	21 ± 3	0.187
*Brain Structure*			
Total brain volume (mL)	1135 ± 101	1025 ± 89	0.002
Total brain volume (%ICV)	75.3 ± 5.9	68.6 ± 5.2	0.001
Intracranial volume (mL)	1517 ± 183	1504 ± 175	0.826
*Blood Pressure and Heart Rate*			
Systolic blood pressure at rest (mmHg)	115 ± 9	121 ± 12	0.086
Diastolic blood pressure at rest (mmHg)	75 ± 9	79 ± 8	0.247
Heart rate at rest (bpm)	71 ± 12	71 ± 7	0.814
Age‐predicted maximal heart rate (bpm)	191 ± 1	165 ± 3	< 0.001
Absolute heart rate during exercise (bpm)^a^	127 ± 9	112 ± 9	< 0.001
Relative heart rate during exercise (%HRmax)^a^	67 ± 5	68 ± 5	0.481
*Treadmill Settings and Perceived Exertion*			
Speed (km/h)^a^	5.9 ± 0.4	4.0 ± 0.8	< 0.001
Grade (%)^a^	5.2 ± 1.1	5.5 ± 2.3	0.666
Post‐exercise rating of perceived exertion^a^	12 ± 1	12 ± 2	0.568

*Note:* Values are means and standard deviations (SD). Exercise heart rate represents a 30 min average. *p* values are calculated from independent *t*‐test for continuous variables and Chi‐square test for categorical variables.

Abbreviation: ICV, intracranial volume.

^a^ Data were available for 17 of the 19 older adults.

### 3.2 Age and Repetition Effects

**FIGURE 2:**
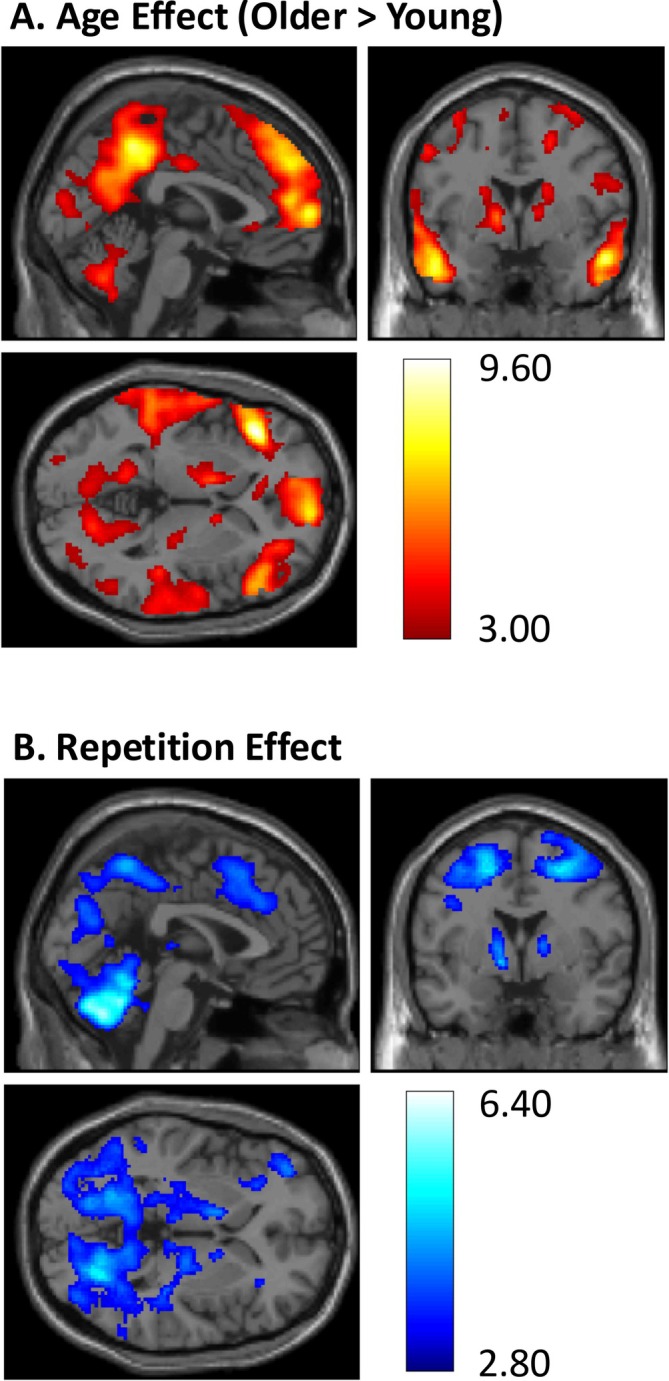
(A) Significant regions showing greater neural switch cost activity (switch minus nonswitch) in older adults compared to young adults. (B) Significant regions showing task repetition‐related decreases in brain activity across both age groups. These regions were identified by whole‐brain voxelwise analysis. Anatomical locations of significant clusters are listed in Table S1.

**FIGURE 3:**
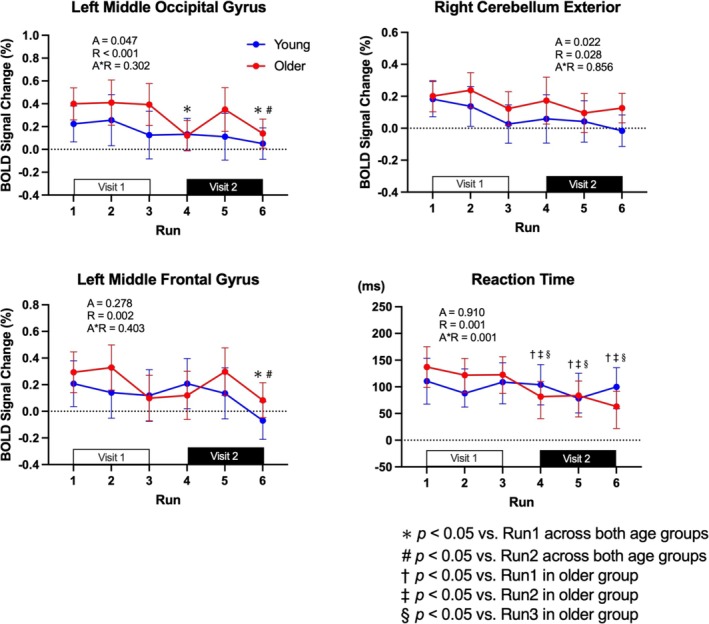
Task repetition effects on neural switch cost activity (switch minus nonswitch) and reaction time switch cost in young and older adults. Blood oxygenation level‐dependent (BOLD) signal changes were extracted from significant clusters identified by the whole brain voxelwise analysis (Figure 2B). Run 1 and Run 4 represent the pre‐intervention measurements collected on visits 1 and 2, respectively, regardless of rest or exercise condition. Values represent estimated marginal means with 95% confidence intervals and *p* values from linear mixed models. Post hoc comparisons were Bonferroni‐corrected. * and # indicate *p* < 0.05 versus Runs 1 and 2 across both groups. †, ‡, and § indicate *p* < 0.05 versus Runs 1, 2, and 3, respectively, in the older group. A, age; R, repetition.

Significant main effects of age and repetition, but no interaction (*p* > 0.05), were observed for switch cost‐related BOLD activation. Compared with young adults, older adults exhibited significantly greater activation in the bilateral superior frontal gyri, bilateral middle temporal gyri, right angular gyrus, left precuneus, temporal pole, and precentral gyrus (Figure 2A, Table S1). No regions showed greater activation in young adults than in older adults (*p* > 0.05). In contrast, reaction time switch cost did not differ significantly by age (*p* = 0.910; Figure 3B), although switch and nonswitch task performance measured by reaction time and error rate was lower in the older group (Table S2).

Across both age groups, task repetition significantly decreased BOLD activation related to switch cost (Figure 2B). Figure 3A shows BOLD signals from significant clusters plotted over six runs, confirming reduced activations in the left middle occipital gyrus, right cerebellar cortex, and left middle frontal gyrus with repetition. No regions showed increased activation with repetition (*p* > 0.05), and there was no significant age × repetition interaction effect (*p* ≥ 0.302).

Significant effects of repetition and an age × repetition interaction were found for reaction time switch cost. Older adults improved their reaction time with repetition, whereas young adults did not (Figure 3). Error rate switch cost was unaffected by age or repetition (*p* ≥ 0.132; Table S2). During both switch and nonswitch tasks, reaction time significantly decreased with repetition in both groups, while switch task error rate significantly declined in older adults.

### 3.3 Exercise Effects

**FIGURE 4:**
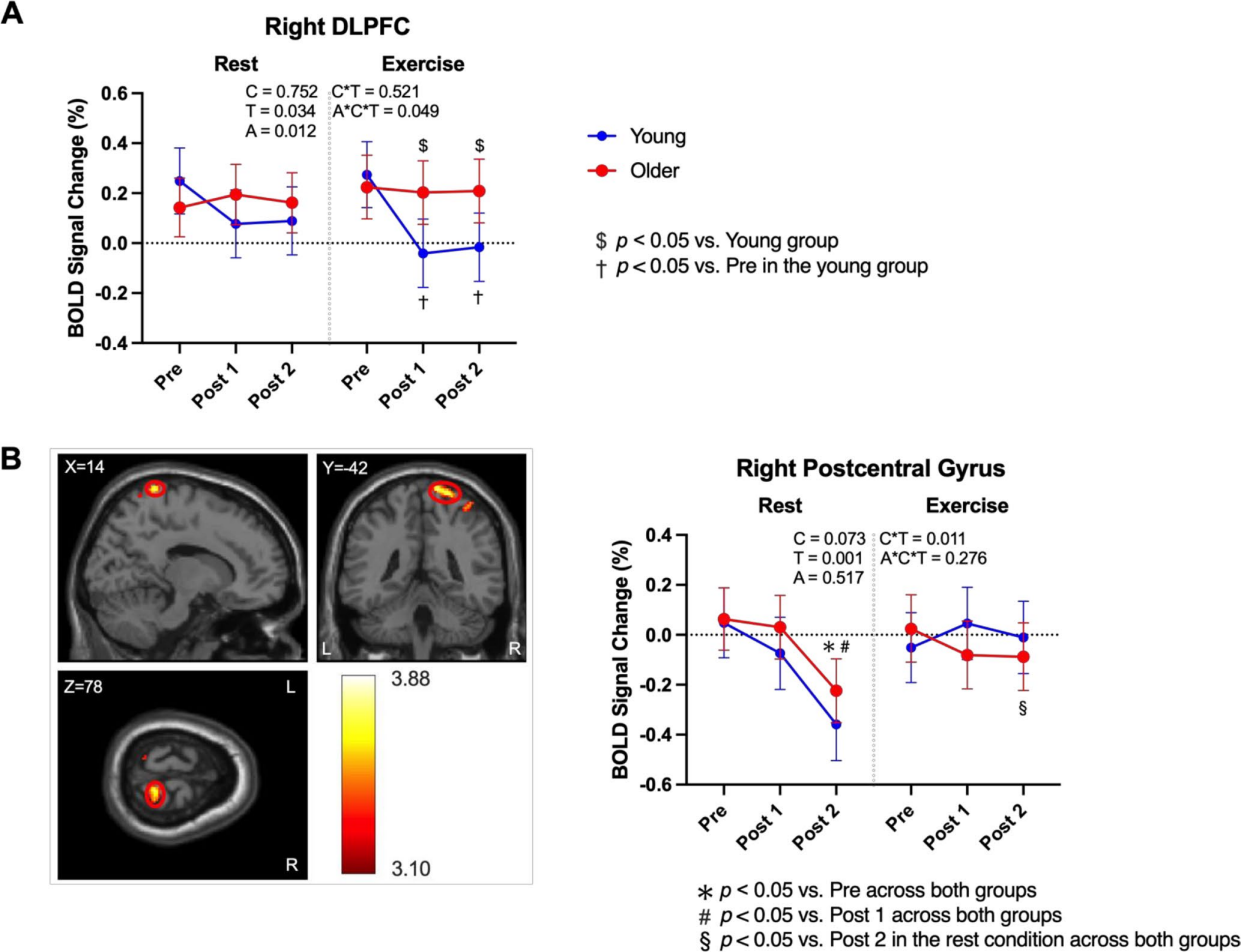
(A) A priori region‐of‐interest analysis showing the acute exercise effects on neural switch cost activity (switch minus nonswitch) in the right dorsolateral prefrontal cortex (DLPFC) in young and older adults. Blood oxygenation level‐dependent (BOLD) signal changes were extracted from the right DLPFC. (B) Whole‐brain analysis showing significant clusters where neural switch cost activity differed between the exercise and rest conditions. The adjacent line graph illustrates differences in BOLD signal changes in the right postcentral gyrus (red circle in brain map) between conditions. Values represent estimated marginal means with 95% confidence intervals and *p* values from linear mixed models. Post hoc comparisons were Bonferroni‐corrected. Models were adjusted for pre‐intervention values in each condition. $ indicates *p* < 0.05 versus Young group. † indicates *p* < 0.05 versus Pre in the young group. * and # indicate *p* < 0.05 versus pre and post 1 across both groups. § indicates post 2 in the rest condition across both groups. A, age; C, condition; T, time.

For the hypothesis‐based ROI analysis, LMM with adjustment for pre‐intervention values showed a significant age × condition × time interaction effect for switch cost‐related BOLD activation in the right DLPFC (η_*p*_^2^ = 0.097). BOLD activation in the region decreased after walking in young adults (Cohen's *d* ≥ 0.517), whereas it remained unchanged after walking in older adults (Figure 4A). BOLD activation in the right DLPFC at post 1 and post 2 after walking was significantly higher in older adults than in young adults (*d* > 0.364). In contrast, no significant condition × time or age × condition × time interaction effects for switch cost‐related BOLD activation in the left DLPFC, bilateral VLPFC, and SMG (*p* ≥ 0.071; Tables 2). Simiraly, no significant condition × time or age × condition × time interaction effects were found for BOLD activation in the bilateral DLPFC, VLPFC, and SMG during the switch and nonswitch tasks (*p* ≥ 0.052; Table S3).

**TABLE 2:** Task‐related brain activity of the predefined regions‐of‐interest in young and older groups.

		Young		Older		*p* (LMM)	
		Rest	Exercise	Rest	Exercise		
		*Switch Cost*					
Left DLPFC (%)	Pre	0.532 (0.339, 0.726)	0.481 (0.288, 0.674)	0.367 (0.194, 0.540)	0.481 (0.294, 0.668)	C = 0.617	C*T = 0.592
	Post 1	0.264 (0.063, 0.464)	0.227 (0.027, 0.427)	0.408 (0.231, 0.586)	0.350 (0.163, 0.537)	T = 0.009	A*C*T = 0.071
	Post 2	0.013 (−0.188, 0.214)	0.348 (0.148, 0.548)	0.403 (0.226, 0.581)	0.260 (0.073, 0.446)	A = 0.214	
Right DLPFC (%)	Pre	0.249 (0.117, 0.381)	0.274 (0.142, 0.406)	0.142 (0.025, 0.260)	0.224 (0.097, 0.352)	C = 0.752	C*T = 0.521
	Post 1	0.077 (−0.059 0.213)	−0.041 (−0.177, 0.096)^b^	0.195 (0.074, 0.315)	0.203 (0.075, 0.330)^a^	T = 0.034	A*C*T = 0.049
	Post 2	0.089 (−0.047, 0.225)	−0.016 (−0.153, 0.120)^b^	0.162 (0.041, 0.282)	0.209 (0.081, 0.336)^a^	A = 0.012	
Left VLPFC (%)	Pre	0.034 (−0.101, 0.170)	−0.028 (−0.164, 0.108)	0.132 (0.011, 0.252)	0.165 (0.033, 0.296)	C = 0.351	C*T = 0.670
	Post 1	−0.023 (−0.163, 0.117)	0.060 (−0.080, 0.200)	0.115 (−0.008, 0.239)	0.188 (0.056, 0.319)	T = 0.849	A*C*T = 0.767
	Post 2	0.025 (−0.115, 0.165)	−0.033 (−0.173, 0.107)	0.065 (−0.058, 0.189)	0.166 (0.035, 0.298)	A < 0.001	
Right VLPFC (%)	Pre	0.197 (0.022, 0.373)	0.199 (0.024, 0.375)	0.196 (0.040, 0.352)	0.211 (0.041, 0.381)	C = 0.655	C*T = 0.834
	Post 1	0.107 (−0.074, 0.289)	0.219 (0.037, 0.400)	0.257 (0.097, 0.417)	0.283 (0.113, 0.453)	T = 0.236	A*C*T = 0.880
	Post 2	0.105 (−0.076, 0.287)	0.110 (−0.071, 0.292)	0.137 (−0.023, 0.297)	0.288 (0.118, 0.457)	A = 0.798	
Left SMG (%)	Pre	0.363 (0.206, 0.520)	0.281 (0.124, 0.438)	0.327 (0.188, 0.466)	0.374 (0.222, 0.526)	C = 0.464	C*T = 0.647
	Post 1	0.272 (0.110, 0.434)	0.290 (0.127, 0.453)	0.359 (0.215, 0.502)	0.379 (0.227, 0.531)	T < 0.001	A*C*T = 0.850
	Post 2	0.088 (−0.075, 0.250)	0.233 (0.071, 0.396)	0.126 (−0.018, 0.269)	0.156 (0.004, 0.308)	A = 0.431	
Right SMG (%)	Pre	0.226 (0.103, 0.349)	0.129 (0.006, 0.253)	0.182 (0.073, 0.291)	0.207 (0.089, 0.326)	C = 0.365	C*T = 0.281
	Post 1	0.011 (−0.116, 0.139)	0.056 (−0.071, 0.183)	0.231 (0.119, 0.343)	0.234 (0.116, 0.353)	T = 0.133	A*C*T = 0.213
	Post 2	−0.053 (−0.180, 0.074)	0.132 (0.004, 0.259)	0.145 (0.033, 0.257)	0.170 (0.051, 0.288)	A = 0.002	

*Note:* Values are estimated marginal means, 95% confidence intervals, and *p* values calculated from the linear mixed model (LMM). The model was adjusted for pre‐intervention values of the corresponding exercise or rest condition.

Abbreviations: A, age; C, condition; DLPFC, dorsolateral prefrontal cortex; SMG, supramarginal gyrus; T, time; VLPFC, ventrolateral prefrontal cortex.

^a^ Indicates *p* < 0.05 versus young group.

^b^ Indicates *p* < 0.05 versus pre in the young group.

**TABLE 3:** Behavioral performance in young and older groups.

		Young		Older		*p* (LMM)	
		Rest	Exercise	Rest	Exercise		
		*Switch Cost*					
Reaction time (ms)	Pre	107.6 (87.3, 127.9)	108.5 (88.2, 128.9)	105.6 (87.6, 123.7)	113.2 (93.5, 133.0)	C = 0.326	C*T = 0.945
	Post 1	91.6 (70.7, 112.6)	94.5 (73.5, 115.5)	94.9 (76.4, 113.4)	108.8 (89.1, 128.6)	T = 0.147	A*C*T = 0.577
	Post 2	105.7 (84.7, 126.7)	100.9 (79.9, 121.8)	82.9 (64.4, 101.5)	102.8 (83.0, 122.5)	A = 0.989	
Error rate (%)	Pre	1.8 (0.2, 3.4)	2.0 (0.4, 3.6)	2.6 (1.1, 4.0)	2.9 (1.3, 4.4)	C = 0.910	C*T = 0.365
	Post 1	1.2 (−0.5, 2.8)	1.1 (−0.5, 2.8)	1.7 (0.2, 3.1)	2.8 (1.2, 4.3)	T = 0.303	A*C*T = 0.879
	Post 2	1.6 (0.0, 3.3)	1.2 (−0.4, 2.9)	2.3 (0.8, 3.8)	0.8 (−0.7, 2.4)	A = 0.159	

*Note:* Values are estimated marginal means, 95% confidence intervals, and *p* values calculated from the linear mixed model (LMM). The model was adjusted for pre‐intervention values of the corresponding exercise or rest condition.

Abbreviations: A, age; C, condition; T, time.

Whole‐brain voxelwise analysis revealed a significant condition × time interaction for switch cost‐related BOLD activation. Significant clusters were identified in the right postcentral gyrus and bilateral superior parietal lobules (SPLs) (Table S4). LMM with adjustment for pre‐intervention values confirmed that walking maintained higher activation in the right postcentral gyrus compared with sitting at both post 1 and post 2 (*d* = 0.535; Figure 4B).

Behavioral outcomes are summarized in Table 3. After adjustment for pre‐intervention values, LMM analyses indicated no significant condition × time or age × condition × time interactions for switch cost performance (*p* ≥ 0.365). Similarly, no significant effects were found for switch or nonswitch task performance (*p* ≥ 0.172; Table S5).

### 3.4 Sex Effects

Whole‐brain voxelwise analyses showed no significant effect of sex × condition × time or sex × repetition interaction on BOLD activation (*p* > 0.05). However, significant main effects of sex were found in voxel‐based analyses (Figure S2). Compared with females, males showed significantly greater activation in the bilateral middle frontal gyrus, supplementary motor area, and middle temporal gyrus, as well as in the left angular gyrus, precuneus, and calcarine cortex, and in the right supramarginal gyrus and anterior insula (Table S6). No regions showed greater activation in males than in females (*p* > 0.05).

### 3.5 Associations Between Cognitive Performance and Brain Activity

**FIGURE 5:**
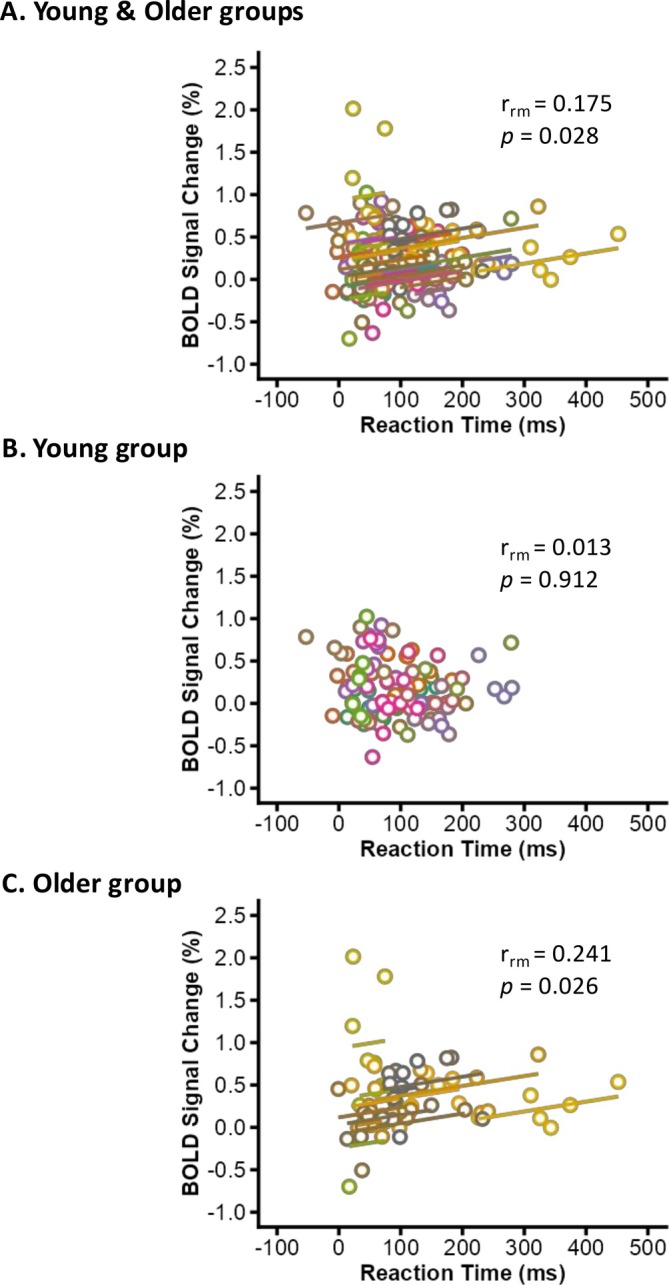
Repeated‐measures correlations between reaction time and blood oxygenation level‐dependent (BOLD) signal changes extracted from significant clusters identified in Figure 2B (left middle occipital gyrus). Panels (A), (B), and (C) correspond to all, young, and older participants, respectively. In the repeated measures correlation plot, different colors represent individual participants. Data points indicate repeated observations, and lines represent individual regression lines. Lines are displayed only when the association was statistically significant.

Across all participants irrespective of exercise or rest condition, reaction time switch cost was positively correlated with BOLD activation in the left middle occipital gyrus, precentral gyrus, and middle frontal gyrus (Figure 5A). When analyzed separately in each age group, the associations between reaction time and BOLD activation were significant only in older adults (Figure 5B,C). No significant correlations were found between error rate switch cost and BOLD activation in any region.

## 4 Discussion

The main findings of this study are as follows. First, older adults exhibited greater and more widespread switch task‐related BOLD activation than young adults, despite slightly reduced behavioral performance. Second, task repetition decreased activation in both age groups, whereas reaction time improved only in older adults. Third, after walking, regional activation in the right DLPFC remained stable in older adults but decreased in young adults, with no corresponding change in behavioral performance. Similarly, activation in the bilateral superior parietal lobules and the right postcentral gyrus remained stable after walking but decreased after sitting in both age groups. Fourth, neither the exercise nor repetition effects on BOLD activation differed by sex, although males showed greater activation and lower error rates than females across conditions and repetitions. Together, these findings suggest that acute aerobic exercise may help preserve task‐related neural recruitment during task switching, with effects largely consistent across age and sex. Below, we discuss potential mechanisms and clinical implications of these results.

### 4.1 Exercise Effects

The predefined ROI analysis revealed significant exercise effects on BOLD activation in the right DLPFC, which has been shown to be involved in task switching [17]. The regional BOLD activation decreased after exercise in the young group, while it was maintained after exercise in the older group. These findings suggest that the acute exercise effects on regional neural recruitment may differ by age. In contrast, BOLD activation in other ROIs decreased after exercise likely due to repetition effects that may have masked modest exercise‐related changes. Accordingly, acute exercise may influence task‐related brain activity in a region‐specific manner. This is supported by whole brain voxelwise analysis demonstrating that BOLD activation in parietal regions (bilateral SPLs and right postcentral gyrus) was maintained after exercise in both age groups. The parietal regions support attentional shifting between perceptual features and task rules [40, 41], indicating that acute aerobic exercise may modulate neural correlates of executive function. The observed maintenance of BOLD activation could potentially reflect nonspecific influences such as global arousal or attentional changes [42]. However, such effects would be expected to produce more widespread changes in BOLD activation, supporting the interpretation that the observed pattern may reflect exercise‐specific modulation of task‐related neural processes.

It should be noted that the BOLD signal reflects the HRF, which captures transient changes in blood oxygenation that are coupled to underlying neuronal activity [18, 19]. To account for potential variability in the timing of the HRF across participants, including age‐ and exercise‐related differences, the present study modeled task‐related responses using the canonical HRF with its temporal derivative. However, blood oxygenation levels are influenced not only by neural activity but also by cerebral blood flow, which delivers oxy‐ and deoxygenated hemoglobin to the neuronal tissue. Oxygen delivery increases substantially during aerobic exercise due to elevated cardiac output and ventilation, and these cardiorespiratory responses may remain elevated for an extended period after exercise depending on intensity and individual fitness levels [43]. Consequently, the altered BOLD activation observed after exercise in the current study may reflect a combination of neural and vascular factors that cannot be disentangled using BOLD imaging alone. In addition, the finding of greater BOLD activation despite comparable or reduced cognitive performance may reflect reduced neural efficiency following exercise. Further research using complementary measures of neural and vascular function will be needed to clarify these possibilities.

A single bout of 30 min moderate‐intensity walking did not acutely improve cognitive flexibility performance compared with sitting in either age group, contrasting with prior studies reporting beneficial effects of acute aerobic exercise on cognitive flexibility [5, 6, 7, 8, 9]. One possible explanation for this discrepancy is the use of pre‐intervention adjustment in the current study, which statistically controlled for inter‐individual differences in baseline cognitive performance, an approach that most prior studies did not incorporate [20]. Consistent with the current study, a previous study [12] found no group‐level cognitive improvement following acute exercise but noted substantial inter‐individual variability in response. Moreover, a recent systematic review [2] reported greater exercise‐related cognitive benefits in individuals with lower baseline cognitive function, emphasizing the value of accounting for baseline performance. Interestingly, indoor walking, similar to the protocol in the current study, did not improve cognitive performance, whereas outdoor walking did [44]. This may suggest that exercise context (e.g., environmental stimulation) may influence the efficacy of acute exercise for enhancing cognitive performance, particularly in older adults.

### 4.2 Task Repetition Effects

To further contextualize the exercise findings in the current study, how task repetition influences neural correlates of executive function across age groups was examined and showed that task repetition decreased BOLD activation during task switching irrespective of age groups. Reduced BOLD activation observed between the first and fourth runs suggests that repetition‐induced learning may persist over the two experimental visits. On the other hand, task repetition improved reaction time only in older adults, consistent with prior studies showing that task repetition did not alter working memory performance in young adults, whereas it facilitated behavioral performance in older adults [33, 34, 45]. These findings suggest that task repetition may increase neural efficiency across age groups, thereby partly contributing to improved behavioral performance in older adults. Furthermore, positive within‐subject correlations were found between reaction time and BOLD signals, indicating that faster responses were associated with lower neural activation across multiple regions, which is consistent with increased processing efficiency. Taken together, these results highlight the importance of considering task repetition and age effects when evaluating the impact of acute exercise on brain function.

### 4.3 Age Effects

The present study showed strong age‐related differences in switch task‐related brain activity between young and older groups. Older adults exhibited higher and more widespread activation than young adults across the frontal, parietal, temporal, and occipital lobes, with reduced switch and nonswitch task performance, but comparable switch cost performance. These findings align with the Scaffolding Theory of Aging and Cognition [28], which proposes that older adults recruit additional brain regions to maintain cognitive performance in the face of age‐related neural decline. While previous studies have reported greater switch cost (i.e., lower performance) in older adults [46, 47], another study has suggested that the impact of age on cognitive flexibility can vary considerably across individuals [48]. Notably, the older participants in the current study were cognitively and physically healthy and free of cardiovascular risk factors (e.g., hypertension) that have been shown to reduce executive function with aging [49].

### 4.4 Sex Effects

A previous review [30] has suggested sex differences in the neural networks underlying executive function, with mixed findings showing higher activation in either males or females. The exploratory results of the current study revealed greater neural switch cost activity in males than in females, indicating that males and females engage different neural strategies during executive function tasks. However, no sex‐related differences were observed in the exercise effects on brain activity, suggesting that acute exercise effects on brain activity underlying executive function are not moderated by sex.

### 4.5 Limitations

First, we employed only a single task switching paradigm to assess cognitive flexibility. While this allowed for a focused interpretation, it limits the generalizability of the findings to other cognitive domains. Moreover, the neural switch cost reflects relative differences between task conditions within the specific experimental structure rather than a pure neural mechanism of task switching. Second, the intervention involved only moderate‐intensity aerobic exercise. Future studies should examine whether similar effects can be observed with lower or higher intensities or with alternative exercise modalities (e.g., resistance training, interval training). Third, the 16‐min duration of the task‐switching session may have contributed to strong learning effects, potentially masking modest benefits of acute exercise. Fourth, the sample size was relatively small, particularly when divided into young and older groups or into males and females, which may have limited statistical power. Therefore, our findings should be replicated in larger and more diverse samples using a similar study design. Fifth, exercise intensity was prescribed using age‐predicted maximal heart rate without a graded exercise test, which may have limited the precision of exercise intensity estimation. Moreover, the prescribed range of 50%–70% of age‐predicted maximal heart rate corresponds to a light‐to‐moderate intensity range according to ACSM criteria [50]. Therefore, the actual exercise intensity may have varied across participants, and such variability may have influenced the magnitude of cognitive and neural effects, although the mean heart rate during exercise corresponded to the moderate‐intensity range. Sixth, unmeasured factors such as regular physical activity and sleep (e.g., frequency, duration, and quality) may have influenced the present results. Lastly, voxelwise whole‐brain analyses corrected for family‐wise error may have limited sensitivity for detecting higher‐order interaction effects (e.g., age × condition × time). Therefore, these voxelwise findings should be interpreted as exploratory.

## 5 Conclusion

Thirty‐minute, moderate‐intensity walking maintained regional BOLD activation involved in task switching in healthy young and older adults compared with sitting. This suggests that acute aerobic exercise may counteract repetition‐related reductions in brain activation, particularly within the frontal and parietal regions supporting executive function. Although behavioral performance did not significantly improve following exercise, the repetition‐related effects may have obscured modest exercise effects. These findings suggest that acute aerobic exercise may influence the brain's neural processes before detectable behavioral changes, particularly in older adults.

## Author Contributions

T.T. and R.A. contributed to the conception and design of experiments. R.A., M.F., D.H., and T.T. contributed to the data acquisition. R.A. and T.T. contributed to the data analyses and data interpretation and drafted the first version of manuscript. D.H., J.S., J.W., and R.Z. provided constructive comments and revised the manuscript. All authors read and approved the final version of this manuscript.

## Funding

This study was supported by the Japan Society for the Promotion of Science (24K20656 to R.A. and 23K24748 and 25K21803 to T.T.) and the 39th Research Grant of Meiji Yasuda Life Foundation of Health and Welfare (to R.A.).

## Conflicts of Interest

The authors declare no conflicts of interest.

## Supporting information


**FIGURE S1:** Flowchart of the study design for the acute aerobic exercise intervention in young and older adults.
**FIGURE S2:** Brain regions showing greater neural switch cost activity (switch > nonswitch) in males compared to females.
