# Supplementary material for: Effects of Acute Aerobic Exercise and Task Repetition on the Neural Correlates of Executive Function in Young and Older Adults: A Crossover fMRI Study
Source: J Magn Reson Imaging. 2026 Jun 10;64(3):751–64. doi: 10.1002/jmri.70329 (PMC13466590; doi:10.1002/jmri.70329)

Supplementary Table 1: Age and repetition effects on whole-brain activity related to switch cost

	MNI coordinate			*T*	Z score
	X	Y	Z		
**Age Effect (Older > Young)**	**Expected voxels per cluster, <k> = 38.152**				
Left Superior Frontal Gyrus	-24	24	50	9.00	Inf
Right Angular Gyrus	54	-58	24	8.90	Inf
Left Precuneus	-10	-50	44	8.86	Inf
Right Middle Temporal Gyrus	54	-2	-20	8.78	7.84
Right Superior Frontal Gyrus	6	62	8	8.76	7.82
Right Angular Gyrus	48	-60	26	8.67	7.76
Left Precuneus	-2	-46	46	8.49	7.63
Right Posterior Orbital Gyrus	36	28	-14	7.89	7.19
Left Temporal Pole	-48	12	-26	7.73	7.07
Left Posterior Cingulate Gyrus	-6	-38	36	7.69	7.03
Left Middle Temporal Gyrus	-56	-6	-18	7.67	7.02
**Repetition Effect**	**Expected voxels per cluster, <k> = 38.152**				
Left Middle Occipital Gyrus	-30	-76	34	5.85	5.54
Right Cerebellum Exterior	20	-62	-24	5.45	5.19
Left Precentral Gyrus	-32	-16	72	5.22	4.99
Left Middle Frontal Gyrus	-38	48	2	4.21	4.08

Inf, Infinity.

Supplementary Table 2: Age and repetition effects on behavioral performance

		Young	Older	*p* -values (LMM)
		Switch Cost		
Reaction time (ms)	Run 1	110.6 (67.6, 153.6)	137.0 (98.8, 175.2)	A = 0.910
	Run 2	97.8 (62.0, 133.5)	121.2 (89.2, 153.2)	R < 0.001
	Run 3	106.7 (68.2, 145.2)	122.1 (87.7, 156.5)	A*R = 0.001
	Run 4	103.9 (66.0, 141.7)	75.2 (40.4, 109.9) † ‡ §	
	Run 5	88.3 (51.2, 125.5)	77.2 (43.5, 110.9) † ‡ §	
	Run 6	97.6 (59.2, 136.0)	56.7 (21.8, 91.6) † ‡ §	
Error rate (%)	Run 1	1.1 (-2.2, 4.5)	5.3 (2.2, 8.3)	A = 0.132
	Run 2	0.4 (-1.8, 2.6)	3.6 (1.6, 5.6)	R = 0.339
	Run 3	1.1 (-1.2, 3.3)	2.4 (0.4, 4.4)	A*R = 0.117
	Run 4	1.5 (-0.4, 3.5)	0.9 (-1.0, 2.8)	
	Run 5	0.9 (-0.6, 2.4)	1.4 (0.0, 2.8)	
	Run 6	0.8 (-0.8, 2.4)	1.5 (0.0, 3.0)	
		Switch Task		
Reaction time (ms)	Run 1	698.6 (625.7, 771.6)	881.0 (816.2, 945.8)	A = 0.002
	Run 2	651.1 (584.6, 717.5)	825.7 (766.4, 885.0) †	R < 0.001
	Run 3	657.4 (590.8, 724.1)	806.5 (747.0, 865.9) †	A*R = 0.005
	Run 4	662.2 (592.6, 731.8)	747.0 (684.0, 810.1) † ‡ §	
	Run 5	609.3 (540.9, 677.7) # $	750.7 (689.2, 812.2) † ‡ §	
	Run 6	610.9 (541.9, 679.9) # $	745.9 (683.8, 808.0) † ‡ §	
Error rate (%)	Run 1	4.2 (0.4, 8.0)	8.1 (4.7, 11.5)	A = 0.354
	Run 2	2.8 (0.3, 5.2)	6.3 (4.1, 8.5)	R = 0.007
	Run 3	3.7 (1.4, 6.0)	4.4 (2.3, 6.4)	A*R = 0.018
	Run 4	5.3 (3.0, 7.7)	3.6 (1.3, 5.8)	
	Run 5	2.8 (0.8, 4.9)	2.9 (1.1, 4.8) † ‡	
	Run 6	2.9 (0.7, 5.1)	3.9 (1.8, 6.0)	
		Nonswitch Task		
Reaction time (ms)	Run 1	588.0 (539.9, 636.1)	744.0 (701.2, 786.7)	A < 0.001
	Run 2	553.6 (507.3, 599.8)	705.2 (664.1, 746.4)	R < 0.001
	Run 3	550.4 (503.7, 597.2)	685.1 (643.5, 726.7)	A*R = 0.115
	Run 4	558.3 (510.0, 606.6)	673.8 (630.0, 717.7)	
	Run 5	521.3 (472.1, 570.4)	675.4 (631.1, 719.8)	
	Run 6	513.0 (461.8, 564.3)	691.2 (644.8, 737.6)	
Error rate (%)	Run 1	3.1 (1.9, 4.3)	2.8 (1.7, 3.9)	A = 0.494
	Run 2	2.3 (1.3, 3.3)	2.7 (1.8, 3.5)	R = 0.048
	Run 3	2.7 (1.7, 3.6)	1.9 (1.1, 2.8)	A*R = 0.584
	Run 4	3.8 (2.5, 5.0)	2.7 (1.5, 3.9)	
	Run 5	1.9 (0.9, 3.0)	1.6 (0.6, 2.6)	
	Run 6	2.2 (1.1, 3.2)	2.4 (1.4, 3.4)	

Values are estimated marginal means, 95% confidence intervals, and *p* -values calculated from the linear mixed model (LMM). # and $ indicate $p < 0.05$ vs. Runs 1 and 3, respectively, in the young group. †, ‡, and § indicate $p < 0.05$ vs. Runs 1, 2, and 3, respectively, in the older group. A, age; R, repetition.

Supplementary Table 3: Task-related brain activity of the predefined regions-of-interest in young and older groups

		Young		Older		*p*-values (LMM)	
		Rest	Exercise	Rest	Exercise		
				Switch Task			
Left DLPFC (%)	Pre	0.494 (0.310, 0.679)	0.465 (0.278, 0.652)	0.524 (0.360, 0.687)	0.565 (0.385, 0.746)	C = 0.805	C*T = 0.943
	Post 1	0.294 (0.103, 0.484)	0.345 (0.152, 0.537)	0.496 (0.327, 0.664)	0.410 (0.230, 0.591)	T = 0.001	A*C*T = 0.388
	Post 2	0.143 (-0.048, 0.333)	0.289 (0.097, 0.481)	0.477 (0.309, 0.645)	0.263 (0.082, 0.444)	A = 0.067	
Right DLPFC (%)	Pre	0.234 (0.093, 0.375)	0.271 (0.133, 0.410)	0.327 (0.203, 0.451)	0.329 (0.194, 0.464)	C = 0.551	C*T = 0.510
	Post 1	0.136 (-0.010, 0.282)	0.087 (-0.056, 0.231)	0.302 (0.175, 0.429)	0.322 (0.187, 0.457)	T = 0.037	A*C*T = 0.532
	Post 2	0.211 (0.066, 0.357)	0.034 (-0.109, 0.177)	0.221 (0.094, 0.348)	0.229 (0.094, 0.364)	A = 0.009	
Left VLPFC (%)	Pre	-0.189 (-0.331, -0.046)	-0.177 (-0.319, -0.036)	-0.075 (-0.201, 0.051)	-0.072 (-0.209, 0.065)	C = 0.502	C*T = 0.802
	Post 1	-0.159 (-0.306, -0.013)	-0.163 (-0.308, -0.017)	-0.093 (-0.222, 0.036)	-0.050 (-0.187, 0.087)	T = 0.961	A*C*T = 0.626
	Post 2	-0.154 (-0.301, -0.007)	-0.047 (-0.193, 0.099)	-0.149 (-0.277, -0.020)	-0.126 (-0.263, 0.011)	A = 0.280	
Right VLPFC (%)	Pre	0.170 (-0.019, 0.359)	0.222 (0.034, 0.409)	0.248 (0.081, 0.414)	0.271 (0.089, 0.453)	C = 0.513	C*T = 0.912
	Post 1	0.254 (0.058, 0.449)	0.272 (0.079, 0.466)	0.262 (0.090, 0.433)	0.267 (0.085, 0.449)	T = 0.195	A*C*T = 0.691
	Post 2	0.216 (0.020, 0.411)	0.213 (0.019, 0.406)	0.019 (-0.152, 0.191)	0.155 (-0.027, 0.337)	A = 0.731	
Left SMG (%)	Pre	0.310 (0.172, 0.447)	0.300 (0.163, 0.438)	0.308 (0.186, 0.430)	0.312 (0.179, 0.445)	C = 0.280	C*T = 0.700
	Post 1	0.202 (0.060, 0.345)	0.357 (0.215, 0.500)	0.289 (0.163, 0.414)	0.281 (0.147, 0.414)	T < 0.001	A*C*T = 0.830
	Post 2	0.102 (-0.041, 0.244)	0.171 (0.029, 0.313)	0.047 (-0.078, 0.173)	0.096 (-0.037, 0.229)	A = 0.646	
Right SMG (%)	Pre	0.170 (0.057, 0.283)	0.164 (0.051, 0.276)	0.161 (0.061, 0.261)	0.167 (0.058, 0.276)	C = 0.559	C*T = 0.856
	Post 1	0.077 (-0.039, 0.194)	0.106 (-0.011, 0.222)	0.170 (0.067, 0.273)	0.226 (0.117, 0.335)	T = 0.045	A*C*T = 0.809
	Post 2	0.025 (-0.092, 0.141)	0.047 (-0.069, 0.164)	0.103 (0.000, 0.206)	0.119 (0.010, 0.228)	A = 0.088	
				Nonswitch Task			
Left DLPFC (%)	Pre	-0.007 (-0.150, 0.136)	-0.011 (-0.154, 0.132)	0.111 (-0.017, 0.238)	0.094 (-0.043, 0.232)	C = 0.542	C*T = 0.203
	Post 1	0.066 (-0.081, 0.214)	0.126 (-0.021, 0.274)	0.044 (-0.087, 0.174)	0.070 (-0.067, 0.208)	T = 0.727	A*C*T = 0.289
	Post 2	0.175 (0.027, 0.322)	-0.045 (-0.193, 0.102)	0.030 (-0.100, 0.161)	0.013 (-0.124, 0.151)	A = 0.848	
Right DLPFC (%)	Pre	-0.007 (-0.128, 0.113)	0.025 (-0.094 0.143)	0.147 (0.039, 0.254)	0.113 (-0.001, 0.227)	C = 0.649	C*T = 0.369
	Post 1	0.049 (-0.076, 0.174)	0.147 (0.025, 0.270)	0.068 (-0.041, 0.178)	0.127 (0.013, 0.241)	T = 0.685	A*C*T = 0.114
	Post 2	0.143 (0.018, 0.267)	0.087 (-0.036, 0.209)	0.020 (-0.089, 0.130)	0.028 (-0.087, 0.142)	A = 0.825	
Left VLPFC (%)	Pre	-0.235 (-0.332, -0.138)	-0.204 (-0.300, -0.107)	-0.188 (-0.274, -0.102)	-0.198 (-0.291, -0.105)	C = 0.751	C*T = 0.198
	Post 1	-0.147 (-0.247, -0.047)	-0.281 (-0.380, -0.181)	-0.184 (-0.272, -0.096)	-0.199 (-0.292, -0.105)	T = 0.555	A*C*T = 0.057
	Post 2	-0.194 (-0.294, -0.094)	-0.063 (-0.162, 0.036)	-0.189 (-0.277, -0.101)	-0.254 (-0.347, -0.160)	A = 0.647	
Right VLPFC (%)	Pre	-0.030 (-0.173, 0.113)	0.020 (-0.122, 0.161)	0.047 (-0.078, 0.173)	0.063 (-0.074, 0.200)	C = 0.860	C*T = 0.653
	Post 1	0.134 (-0.014, 0.282)	0.047 (-0.099, 0.193)	0.004 (-0.125, 0.133)	-0.013 (-0.151, 0.124)	T = 0.620	A*C*T = 0.052
	Post 2	0.119 (-0.029, 0.266)	0.120 (-0.026, 0.266)	-0.118 (-0.248, 0.011)	-0.130 (-0.267, 0.008)	A = 0.053	
Left SMG (%)	Pre	-0.040 (-0.176, 0.097)	-0.009 (-0.146, 0.128)	-0.022 (-0.143, 0.099)	-0.042 (-0.175, 0.090)	C = 0.990	C*T = 0.672
	Post 1	-0.068 (-0.209, 0.073)	0.011 (-0.130, 0.153)	-0.074 (-0.199, 0.050)	-0.079 (-0.211, 0.053)	T = 0.857	A*C*T = 0.752
	Post 2	0.044 (-0.097, 0.185)	-0.080 (-0.221, 0.061)	-0.083 (-0.207, 0.042)	-0.041 (-0.173, 0.092)	A = 0.464	
Right SMG (%)	Pre	-0.040 (-0.138, 0.058)	0.012 (-0.086, 0.111)	-0.023 (-0.110, 0.064)	-0.032 (-0.127, 0.063)	C = 0.472	C*T = 0.150
	Post 1	0.079 (-0.022, 0.180)	0.025 (-0.076, 0.127)	-0.069 (-0.159, 0.020)	0.000 (-0.095, 0.094)	T = 0.566	A*C*T = 0.169
	Post 2	0.102 (0.000, 0.203)	-0.099 (-0.201, 0.002)	-0.051 (-0.140, 0.038)	-0.042 (-0.137, 0.052)	A = 0.111	

Values are estimated marginal means, 95% confidence intervals, and *p*-values calculated from the linear mixed model (LMM). The model was adjusted for pre-intervention values of the corresponding exercise or rest condition. $ indicates $p < 0.05$ vs. young group. † indicates $p < 0.05$ vs. pre in the young group. A, age; C, condition; DLPFC, dorsolateral prefrontal cortex; SMG, supramarginal gyrus; VLPFC, ventrolateral prefrontal cortex; T, time.

Supplementary Table 4: Exercise effects on whole-brain activity related to switch cost

	MNI coordinate			*T*	Z score
	X	Y	Z		
**Exercise Effect**	**Expected voxels per cluster, <k> = 39.554**				
Right Postcentral Gyrus	14	-42	78	3.76	3.64
Left Superior Parietal Lobule	-22	-50	72	3.75	3.63
Right Superior Parietal Lobule	16	-56	70	3.58	3.48

Supplementary Table 5: Behavioral performance in young and older groups

		Young		Older		*p*-values (LMM)	
		Rest	Exercise	Rest	Exercise		
Switch Task							
Reaction time (ms)	Pre	743.7 (713.5, 773.9)	747.0 (717.3, 776.8)	761.2 (734.6, 787.7)	764.6 (735.3, 793.9)	C = 0.625	C*T = 0.497
	Post 1	701.0 (669.9, 732.1)	691.6 (660.9, 722.3)	735.0 (708.0, 762.0)	736.8 (707.5, 766.0)	T < 0.001	A*C*T = 0.809
	Post 2	710.6 (679.6, 741.6)	686.8 (656.2, 717.4)	727.2 (700.1, 754.2)	719.2 (689.9, 748.4)	A = 0.032	
Error rate (%)	Pre	5.0 (3.3, 6.8)	5.2 (3.4, 6.9)	5.6 (4.0, 7.1)	5.6 (4.0, 7.3)	C = 0.775	C*T = 0.464
	Post 1	3.3 (1.5, 5.1)	2.9 (1.1, 4.7)	4.0 (2.4, 5.6)	5.2 (3.5, 6.9)	T = 0.009	A*C*T = 0.871
	Post 2	4.0 (2.2, 5.8)	3.1 (1.3, 4.9)	4.4 (2.8, 6.0)	3.4 (1.8, 5.1)	A = 0.153	
Nonswitch Task							
Reaction time (ms)	Pre	639.3 (616.7, 661.9)	641.1 (619.1, 663.1)	651.4 (631.5, 671.3)	651.1 (629.6, 672.5)	C = 0.196	C*T = 0.172
	Post 1	611.1 (587.9, 634.3)	598.9 (576.3, 621.6)	636.1 (616.0, 656.3)	627.6 (606.2, 649.1)	T < 0.001	A*C*T = 0.706
	Post 2	608.4 (585.2, 631.6)	588.8 (566.2, 611.4)	640.3 (620.1, 660.4)	616.1 (594.7, 637.6)	A = 0.028	
Error rate (%)	Pre	3.2 (2.4, 4.1)	3.2 (2.3, 4.1)	3.0 (2.3, 3.8)	2.8 (1.9, 3.6)	C = 0.797	C*T = 0.956
	Post 1	2.1 (1.2, 3.0)	1.8 (0.9, 2.7)	2.3 (1.5, 3.1)	2.4 (1.6, 3.3)	T = 0.004	A*C*T = 0.623
	Post 2	2.4 (1.5, 3.3)	1.9 (1.0, 2.8)	2.1 (1.3, 2.9)	2.7 (1.8, 3.5)	A = 0.687	

Values are estimated marginal means, 95% confidence intervals, and *p*-values calculated from the linear mixed model (LMM). The model was adjusted for pre-intervention values of the corresponding exercise or rest condition. A, age; C, condition; T, time.

Supplementary Table 6: Sex effects on whole-brain activity related to switch cost

	MNI coordinate			*T*	Z score
	X	Y	Z		
**Sex Effect (Males > Females)**	**Expected voxels per cluster, <k> = 47.997**				
Left Angular Gyrus	-26	-76	44	6.09	5.74
Left Middle Frontal Gyrus	-40	38	24	5.86	5.55
Left Supramarginal Gyrus	-46	-50	44	5.66	5.38
Left Supprementary Motor Cortex	-2	6	50	4.90	4.71
Left Calcarine Cortex	-4	-88	-4	4.41	4.26
Left Precuneus	-4	-46	52	4.34	4.20
Right Supramarginal Gyrus	66	-38	26	4.06	3.94
Right Middle Temporal Gyrus	56	-60	0	3.99	3.88
Right Cerebellum Exterior	22	-86	-24	3.79	3.70

Supplementary Figure 1

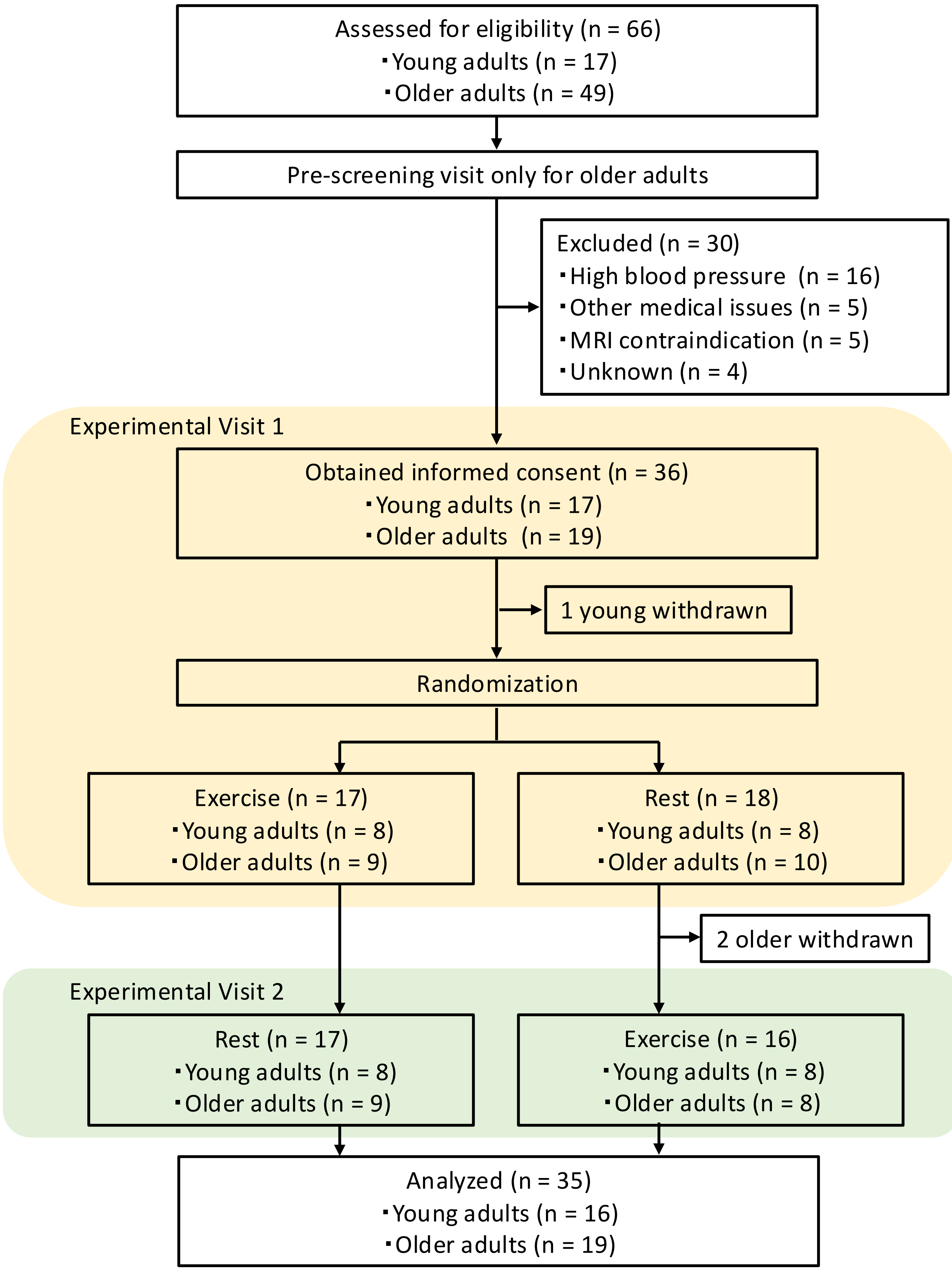

Sex Effect (Male > Female)

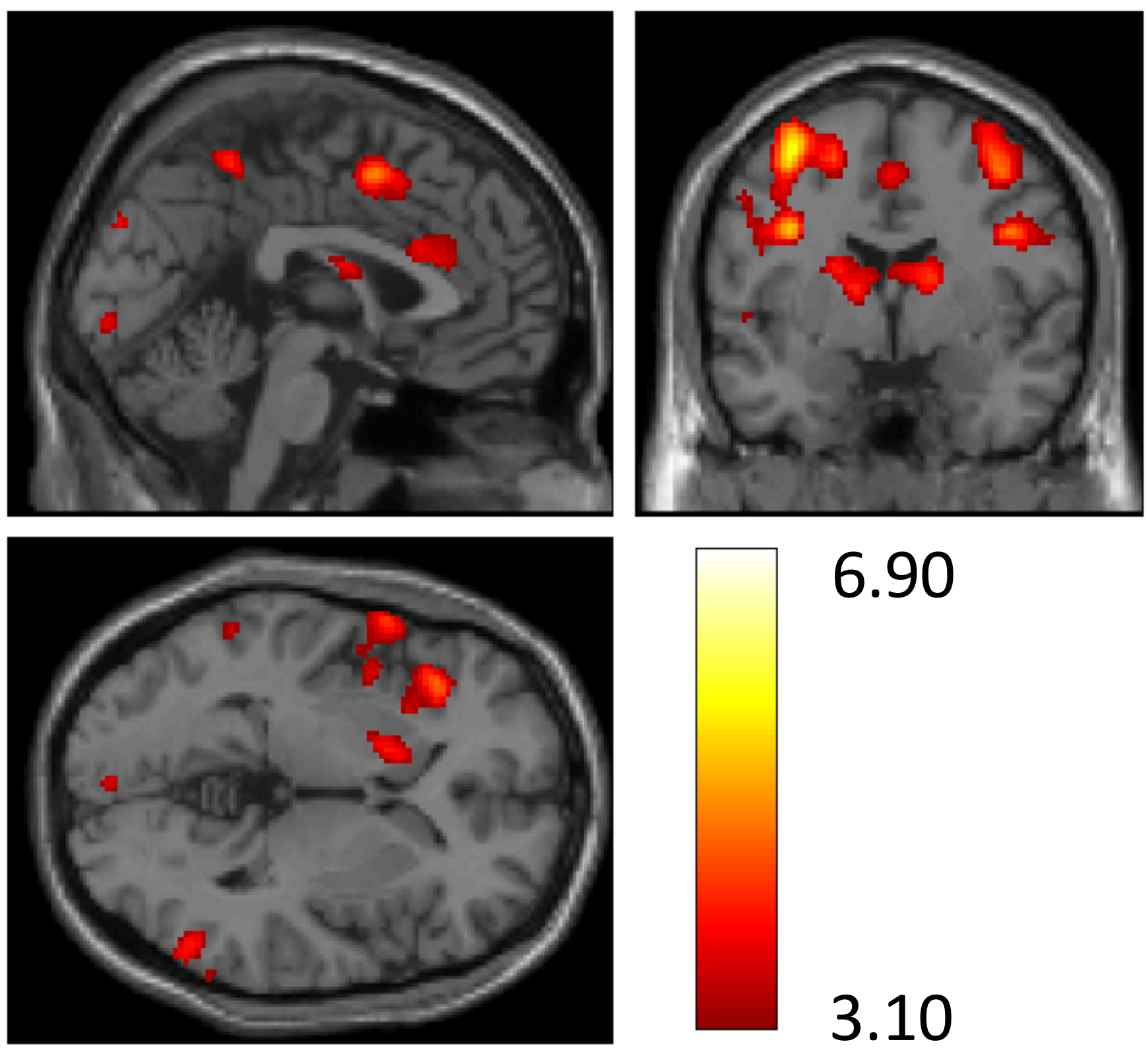

Supplement: Supplementary file 1 [file JMRI-64-751-s001.pdf]
